# The small G protein Arl5 contributes to endosome-to-Golgi traffic by aiding the recruitment of the GARP complex to the Golgi

**DOI:** 10.1242/bio.201410975

**Published:** 2015-03-20

**Authors:** Cláudia Rosa-Ferreira, Chantal Christis, Isabel L. Torres, Sean Munro

**Affiliations:** MRC Laboratory of Molecular Biology, Cambridge CB2 0QH, UK

**Keywords:** Membrane traffic, Golgi apparatus, Arf family GTPase

## Abstract

The small G proteins of the Arf family play critical roles in membrane trafficking and cytoskeleton organization. However, the function of some members of the family remains poorly understood including Arl5 which is widely conserved in eukaryotes. Humans have two closely related Arl5 paralogues (Arl5a and Arl5b), and both Arl5a and Arl5b localize to the trans-Golgi with Arl5b being involved in retrograde traffic from endosomes to the Golgi apparatus. To investigate the function of Arl5, we have used *Drosophila melanogaster* as a model system. We find that the single Arl5 orthologue in *Drosophila* also localizes to the trans-Golgi, but flies lacking the Arl5 gene are viable and fertile. By using both liposome and column based affinity chromatography methods we find that Arl5 interacts with the Golgi-associated retrograde protein (GARP) complex that acts in the tethering of vesicles moving from endosomes to the trans-Golgi network (TGN). In *Drosophila* tissues the GARP complex is partially displaced from the Golgi when Arl5 is absent, and the late endosomal compartment is enlarged. In addition, in HeLa cells GARP also becomes cytosolic upon depletion of Arl5b. These phenotypes are consistent with a role in endosome-to-Golgi traffic, but are less severe than loss of GARP itself. Thus it appears that Arl5 is one of the factors that directs the recruitment of the GARP complex to the trans-Golgi, and this function is conserved in both flies and humans.

## INTRODUCTION

Specific transport of cargo between distinct intracellular membranes relies on spatial landmarks provided by a sophisticated network of phosphoinositides and small G proteins of the Rab and Arf families (Di Paolo and De Camilli, 2006; Gillingham and Munro, 2007; Pfeffer, 2013). Fusion of vesicular cargo carriers to an acceptor membrane occurs via the assembly of particular sets of membrane-bound SNARE proteins found on both compartments (Hong and Lev, 2014). However upstream of SNARE assembly a process called tethering is thought to help capture the vesicle on the correct compartment. Tethering factors are typically large coiled-coil proteins and multisubunit complexes that enable long-range contact between transport carriers and acceptor membranes, and potentially then act to initiate SNARE assembly (Whyte and Munro, 2002; Wong and Munro, 2014; Yu and Hughson, 2010).

Several of the multisubunit tethering complexes comprise a family initially called quatrefoil but subsequently renamed CATCHR (Complex Associated with Tethering Containing Helical Rods) (Whyte and Munro, 2002; Yu and Hughson, 2010). The GARP complex (Golgi-associated retrograde protein), belongs to this family and participates in the delivery to the trans-Golgi network (TGN) of retrograde carriers derived from endosomes in a wide range of eukaryotes (Bonifacino and Hierro, 2011; Conibear and Stevens, 2000). More specifically, it has been shown that the GARP complex, composed of the four proteins Vps51, Vps52, Vps53 and Vps54, is required for the retrieval of late-Golgi SNAREs, TGN46 and receptors for precursors of lysosomal hydrolases such as the mannose 6-phosphate receptors (MPRs), to the TGN (Pérez-Victoria et al., 2008; Quenneville et al., 2006). Depletion of GARP components by RNAi results in missorting of the acid hydrolase cathepsin D and the accumulation of enlarged endosomal compartments (Luo et al., 2011; Pérez-Victoria et al., 2008; Pérez-Victoria et al., 2010).

Like other tethering complexes, it is likely that recruitment of the GARP complex to the TGN is governed by small G proteins of the Rab and Arf families. In the budding yeast *Saccharomyces cerevisiae*, Arl1 and Ypt6p (the ortholog of human Rab6) were found to interact with GARP subunits, with deletion of at least Ypt6 causing the complex to be substantially delocalized (Siniossoglou and Pelham, 2001). However *S. cerevisiae* has lost several members of the Rab and Arf families that were present in the last eukaryotic common ancestor (LECA), and because this yeast has been the major model system for genetic studies of membrane traffic the role of these ‘dispensable’ members of the families has only recently started to emerge (Donaldson and Jackson, 2011). Of the five LECA Arfs that have been lost in budding yeasts, Arl3, Arl6 and Arl13 are involved in cilia and flagella, and Arl8 in lysosome function (Gillingham and Munro, 2007). The fifth is Arl5 with there being two clear Arl5 paralogues in humans, Arl5a and Arl5b, with a third related gene *ARL5C* being expressed at such low levels that it may be a pseudogene. Recently it was shown that both Arl5a and Arl5b are on the Golgi, and that knock-down of Arl5b perturbs endosome-to-Golgi retrograde transport (Houghton et al., 2012). However Arl5b has no reported effectors and so its precise role remains unclear. To investigate Arl5 in more depth we examined the role of the single Arl5 ortholog present in *Drosophila* by using genetics to generate a null allele, and affinity chromatography to seek effectors. Although the gene is not required for viability or fertility we do find that there are morphological changes in the endosomal system. In addition Arl5 appears to contribute to recruitment of the GARP complex to the Golgi in both *Drosophila* and human cells. Our finding supports a model by which Arl5 participates in retrograde traffic from endosomes to the TGN through recruitment of the GARP complex.

## Materials and Methods

### *Drosophila* stocks, mutagenesis and genetics

**Fig. 1.**
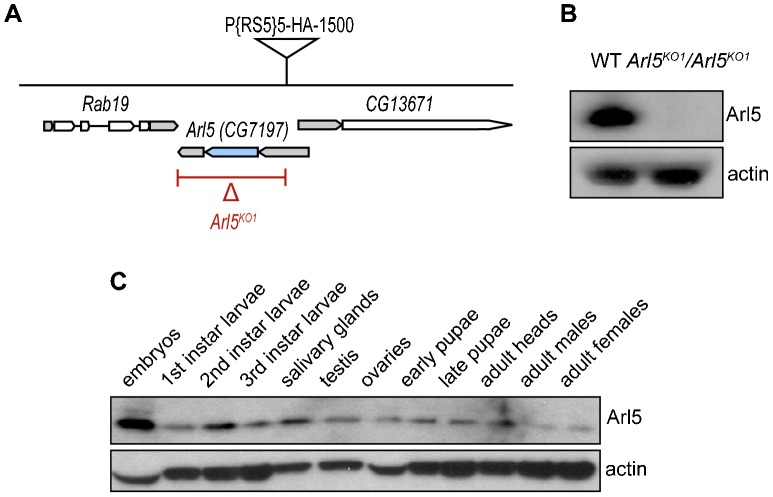
Generation of a null allele for the gene CG7197/Arl5. (A) Schematic illustration of the genomic region containing the *Arl5* and its flanking genes. The P element P{RS5} represented was excised imprecisely, generating a null mutant lacking the coding region of the *Arl5* gene (CG7197) as well as its 3′UTR and part of the 5′UTR (depicted by the red line below the *Arl5* gene). The grey areas indicate untranslated regions (UTR) of the illustrated genes and the blue region represents the coding region of the *Arl5* gene. (B) Arl5 is absent from the *Arl5^KO1^* mutant line. Immunoblot of adult fly lysates from OregonR (WT) and *w1118*; *Arl5^KO1^*/*Arl5^KO1^* probed with a rabbit polyclonal antibody against Arl5 and with an anti-β-Actin polyclonal antibody, used as a loading control. (C) Immunoblot of lysates corresponding to a range of fly tissues and to various stages of development were probed with antibodies against Arl5 and Actin (used as a loading control) revealed that Arl5 is widely expressed.

Unless otherwise stated, fly stocks were maintained at 25C and *w1118* was used as the wild-type control. Mutagenesis of the *Arl5* gene (CG7197) was done by imprecise excision of the P element P{RS5}5-HA-1500 located at its 5′UTR as illustrated in Fig. 1A. The stock was obtained from Bloomington Stock Centre, confirmed by PCR of the flanking sequences and outcrossed to *y1, w1118* for three generations prior to excision, Detection of genomic deletions was then performed by PCR. Cloning and sequencing of the *Arl5^KO1^* allele confirmed the deletion of the entire coding region including the 3′ UTR and part of the 5′ UTR (the 1050 base pairs 8298517–8299566 inclusive replaced by 34 base pairs of uncertain origin).

Arl5-GFP transgenic lines were obtained by germ line transformation (BestGene Inc) of Arl5 cloned into p-UAST and subsequent mapping with *w1118; If/CyO; MKRS/TM6B*. Vps52-GFP was generated by subcloning into the vector pUASP and germline transformation performed by BestGene Inc. The other transgenes used were *P{w^+^*, UASp-YFP.dRab7*}/SM5*, obtained from the Bloomington Stock Centre; *P{w^+^, Sgs3-DsRed}*, a gift from Andrew Andres; and *P{w^+^*, α*tub-GFP-Lerp}* was kindly provided by Julie Brill (Burgess et al., 2012). Act5C-GAL4 was used to drive the expression of all transgenes.

### Cell culture and RNAi

HeLa and COS-7 cells were grown at 37°C in DMEM with penicillin and streptomycin, supplemented with 10% heat inactivated fetal calf serum (FCS). To have a higher proportion of transfected cells expressing transgenes of interest at low levels, plasmid DNA transfection was performed with polyethylenimine (PEI, Polysciences) at working concentration of 1.25 µg/µl, except for the construct myc-Vps54 (human isoform 2 NM_001005739), which was transfected with Lipofectamine LTX (Life Technologies). Knockdown experiments were performed by transfecting HeLa cells with 50 nM of ON-TARGETplus siRNAs (Thermo Scientific) twice (interval of 24 h) with Oligofectamine (Invitrogen) according to manufacture's instructions and subsequent cell harvesting/analysis 54 h after the first transfection. If necessary, cells were also transfected with plasmid DNA 6 h after the first siRNA transfection. The siRNAs used were Arl5a siRNA5 5′-UGGAUGAUGUCACGACUUA-3′ (J-012408-05) for Arl5a, Arl5b siRNA18 5′-CAGCUGAAAUCUCGAAAUA-3′ for Arl5b (J-017861-18) and Nontargeting siRNA #1 (D-001810-01) as negative control.

### Site-directed mutagenesis and RT-PCR

A construct of Arl5b resistant to siRNA treatment (Arl5b-GFP^Res^) was made by introducing 4 silent mutations into the region of Arl5b-GFP targeted by Arl5b siRNA18. For the RT-PCR reactions, equal amounts of total RNA for each sample, purified with SV Total RNA Isolation System (Promega), were used for reverse transcription with random primers mix and the ImProm-II Reverse Transcription System (Promega), as advised by the manufacturer. Equivalent quantities of the cDNA synthesized were further amplified by PCR with gene-specific primers.

### Affinity chromatography

GST-Arl5 T30N and GST-Arl5 Q70L were produced in *Escherichia coli* BL21-GOLD (DE3) and used for large-scale affinity chromatography of cytosol prepared from adult fly heads. *Drosophila* heads were extracted by snap freezing wild-type adult flies (age 1–4 days). Heads were separated from the remaining bodies by quickly vortexing frozen flies and collected by filtering them with liquid nitrogen through a sieve with an aperture size of 710 µm (diameter) on top of a sieve of 425 µm (Van Gelder et al., 1995) (https://www.youtube.com/watch?v = Dn2mp9OKhFc). Affinity chromatography was performed with GST fusions from 1 litre bacterial cultures and lysate from 360 µl fly heads. Bacterial lysis and immobilization of GST fusions on beads was performed as described previously but with 1% CHAPS (Rosa-Ferreira and Munro, 2011). Affinity purification of effectors from *Drosophila* S2 cell lysates using Arl5 GDP-locked and GTP-locked forms on liposomes was performed using His-tagged G protein and liposomes containing Ni-NTA-labelled lipid as described previously for Arl1 (Christis and Munro, 2012).

### Protein extracts and immunoblotting

Whole adult flies, dissected ovaries or whole L3 larvae were lysed in equivalent amounts of SDS-PAGE sample buffer. Insoluble debris was removed by brief centrifugation. HeLa cells, previously washed in ice-cold PBS were directly dissolved in SDS-PAGE sample buffer. Protein extracts were separated in 4–20% Tris-Glycine gels (Invitrogen) and transferred to PVDF membranes for subsequent probing with primary and HRP-conjugated secondary (Santa Cruz Biotechnology) antibodies, detected by chemiluminescence (ECL, Amersham).

### Antibodies immunostaining and imaging

L3 larval salivary glands were fixed in 4% formaldehyde before blocking and permeabilization in PBS, 0.3% Triton X-100, 20% FCS. Ovaries from 2–3 days old females were fixed in 8% formaldehyde and blocked/permeabilized in PBS, 0.1% Tween-20, 20% FCS. Tissue culture cells were fixed in 4% formaldehyde, 24–48 h post-transfection. Blocking and permeabilization was performed in PBS, 0.5% Triton X-100, 20% FCS. Labelling was done by sequential incubation with primary and secondary Alexa Fluor-conjugated (Invitrogen) antibodies prior to mounting in Vectashield (Vector Laboratories).

The antibodies used for probing *Drosophila* proteins were rabbit anti-dGM130 (ab30637, Abcam), rabbit anti-beta Actin (ab8227, Abcam), mouse anti-GFP (11 814 460 001, Roche), rabbit anti-Rab7 (Tanaka and Nakamura, 2008), rabbit anti-AP1 (Hirst et al., 2009), goat anti-dGolgin-245 and rabbit anti-Arl5, the latter two generated in our lab. For mammalian cells, the antibodies used were sheep anti-TGN46 (AHP500G, AbD Serotec), mouse anti-c-Myc (9E10, M4439, Sigma), and rabbit anti-GFP (A-11122, Life Technologies). Images were obtained with a Zeiss LSM 780 confocal microscope.

Fiji (ImageJ) was used for image analysis and subsequent quantitation. To calculate the average fluorescence intensity of the Golgi fraction of Vps52-GFP in comparison to that of the cytoplasmic fraction in follicle cells, a mask of the Golgi pool was created for each image, using endogenous dGolgin-245 specific fluorescent signal. This mask and nuclear regions, obtained with a specific threshold level for the dGolgin-245 fluorescence, were subtracted from each image to create a cytoplasm mask. Both Golgi and cytoplasm masks were further used to calculate the average pixel intensity for each region. Fluorescence intensity values were normalized to wild-type for each image and the average plotted, where scale bars are the values of the standard error of the mean. To calculate the average fluorescence of YFP-Rab7 or GFP-LERP positive puncta, masks of those puncta were created so that the smallest positive structures observed in wild-type images were entirely selected, to exclude any cytoplasmic staining. Fluorescence intensity values were normalized to the *Arl5^KO1^* mutant for each image and the average plotted, where scale bars are the values of the standard error of the mean. Determination of the percentage of HeLa cells with myc-Vps54 at the TGN was done by randomly selecting cells from each experimental condition that were expressing myc-Vps54 only or myc-Vps54 and Arl5b-GFP^Res^. It was considered that cells were expressing myc-Vps54 when the average pixel value of an individual cell was equal or greater than 30.

## Results

### Generation of a *Drosophila Arl5* null mutant

*Drosophila* have a single Arl5 ortholog encoded by the gene CG7197, which we will refer to here as *Arl5*. To generate a null allele for this gene we used imprecise P element excision with the transposase Δ2–3 and a line carrying a copy of the transposon P{RS5}, inserted at the 5′ UTR of the *Arl5* gene, as illustrated in Fig. 1A. One of the lines recovered with loss of the P element marker contained a deletion that removed the entire coding region of the *Arl5* gene, but not the flanking genes, and we will refer to this null allele as *Arl5^KO1^*. We also generated an antiserum against *Drosophila* Arl5 which confirmed that no Arl5 protein was detected in *Arl5^KO1^* homozygous flies (Fig. 1B). Immunoblots of extracts from several fly tissues representing a range of development stages also confirmed that Arl5 is widely expressed (Fig. 1C) as had been suggested by the relatively ubiquitous expression of the mRNA reported in FlyAtlas (Chintapalli et al., 2007). However, despite this ubiquitous expression, homozygous *Arl5^KO1^*/*Arl5^KO1^* flies were viable and appeared grossly normal with no detectable defects in fertility.

### Arl5 localizes to the TGN but is not required for AP-1-dependent secretory granule formation

**Fig. 2.**
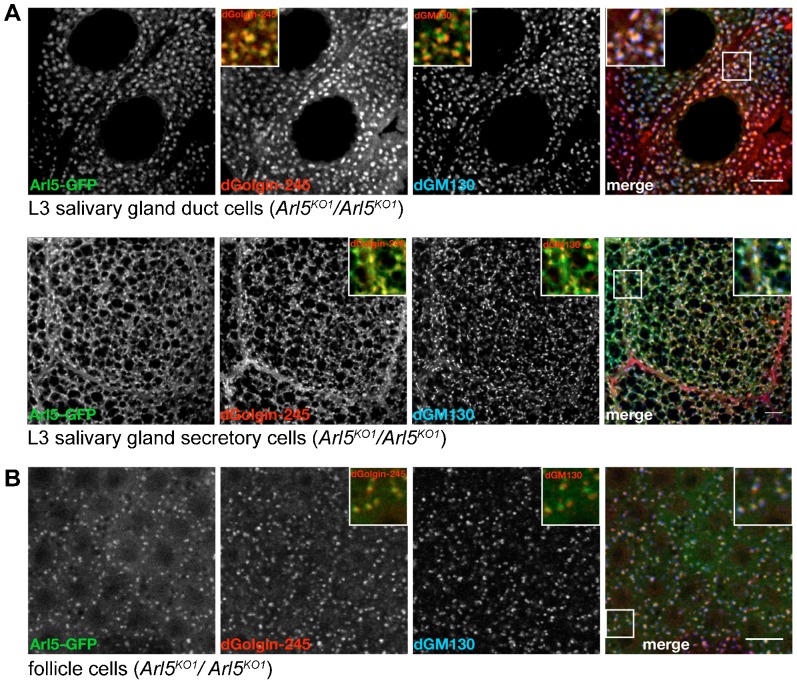
Arl5-GFP localizes to the TGN in fly tissues. (A) Confocal micrographs of the duct cells and secretory cells of the L3 larval salivary gland from flies expressing Arl5-GFP under the control of the Act5C promoter in a *Arl5^KO1^*/*Arl5^KO1^* mutant background. In both tissues, Arl5-GFP was found contiguous to the cis-Golgi marker dGM130 (blue) and overlapped with the trans-Golgi marker dGolgin-245 (red). The insets show magnified images of the area indicated by the boxed regions and correspond to merge images of Arl5-GFP (green) and dGolgin-245 staining (red); of Arl5-GFP (green) and dGM130 staining (red) or of all the 3 channels, respectively. Scale bars are 10 µm. (B) Confocal micrographs of epithelial follicle cells from egg chambers at stage 10 of development expressing Arl5-GFP under the control of the Act5C promoter in a *Arl5^KO1^*/*Arl5^KO1^* mutant background. Again, Arl5-GFP was found contiguous to the cis-Golgi marker dGM130 (blue) and overlapped with the trans-Golgi marker dGolgin-245 (red); insets as in (A). Scale bar 10 µm.

To gain further insight into the role of Arl5 in *Drosophila* we next determined the intracellular location of a GFP-tagged form of the protein. Randomly inserted transgenic UAS-Arl5-GFP lines were generated, and Arl5-GFP was expressed ubiquitously under control of Act5C-GAL4 in flies homozygous for the *Arl5^KO1^* mutation. We first examined the localization of Arl5-GFP in L3 larval salivary glands, a specialized secretory tissue that has been used to study a range of membrane traffic processes. In both the secretory and the duct cells of the salivary gland Arl5-GFP was adjacent to the cis-Golgi marker dGM130 but partially overlapped with the trans-Golgi marker dGolgin-245, thus indicating a location on the trans side of the Golgi (Fig. 2A). Recently, it was shown that Arl1, another member of the Arl family which localizes to the trans-Golgi, plays a role in the salivary gland in both glue granule formation and also the recruitment of the AP-1 clathrin adaptor to the TGN (Torres et al., 2014). However we found no obvious alterations in size and number of granules or in the localization of endogenous AP-1 in the *Arl5^KO1^* mutant, indicating that Arl5 is not required for the sorting mechanisms involved in glue granule biogenesis (supplementary material Fig. S1A,B). We also examined the localization of Arl5-GFP in ovarian follicle cells. Similar to what we observed in salivary glands, in *Arl5^KO1^* mutant follicle cells Arl5-GFP was found adjacent to dGM130 and partially overlapping with dGolgin-245 (Fig. 2B), again suggesting a function at the trans site of the Golgi.

### Affinity chromatography with Arl5 reveals an interaction with the GARP complex

**Fig. 3.**
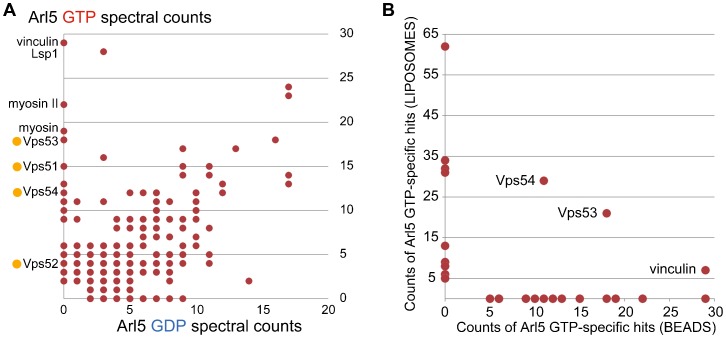
Affinity chromatography of cell extracts with Arl5. (A) Comparison of the spectral counts for proteins isolated from lysates prepared from *Drosophila* heads by affinity chromatography with GST tagged forms of GTP-locked or GDP-locked Arl5. Abundant GTP-specific interactors are labeled, ochre dots indicating GARP subunits. Full list of bound proteins is in supplementary material Table S1. (B) Comparison of the spectral counts for the top twenty proteins found exclusively with the GTP-form of Arl5 in the affinity purification shown in (A) versus the same from an affinity purification performed with Arl5 bound to liposomes. Proteins in both datasets are indicated. See supplementary material Table S2 for the full list of proteins from the liposome purification.

To identify effectors that Arl5 recruits to the Golgi we used two different affinity chromatography methods based on versions of Arl5 carrying mutations that have been shown in other members of the Arf family to lock the protein in the GDP- (inactive) or GTP-bound (active) state (Christis and Munro, 2012; Dascher and Balch, 1994; Houghton et al., 2012). In the first approach GST fusions to the Arl5 forms were immobilized on Sepharose beads and used to isolate proteins from lysates prepared from adult heads. After washing, the bound proteins were eluted with high salt, separated on a protein gel, digested with trypsin, and the resulting peptides sequenced by tandem mass spectrometry. The number of spectra obtained for each protein was used as an approximate measure of abundance and used to compare binding to the GTP- and GDP-bound forms of Arl5 (Fig. 3A). Amongst the more abundant proteins that bound specifically to Arl5-GTP were the *Drosophila* orthologues of the four subunits of the GARP complex (CG15087/Vps51, CG7371/Vps52, CG3338/Vps53, and CG3766/Vps54 or Scat)). The other abundant interactors were mostly proteins that interact with the actin cytoskeleton but these are relatively common contaminants in affinity purifications (Mellacheruvu et al., 2013).

We also used a second approach to identify Arl5 effectors which was a liposome-based method where the His-tagged G protein is used to coat liposomes which are then used to isolate proteins from cytosolic lysates of the *Drosophila* S2 cell line. This latter method has been found to work well for Arf1 and Arl1 from *Drosophila* and may be better for any effectors that also interact with the adjacent lipid bilayer (Christis and Munro, 2012). Proteins bound to liposomes coated with Arl5 forms were identified by mass spectrometry as before. Again, subunits of GARP were amongst the most abundant proteins specific for the GTP-form of Arl5. When we compared the top twenty GTP-specific proteins from the two datasets then only three proteins were in common, two of which were GARP subunits (Fig. 3B). The third was the actin binding protein vinculin, although the significance of this is not clear. In a recent study of *Drosophila* Rab effectors we found evidence for the existence of a second form of the GARP complex in which Vps54 is replaced by a distantly related protein CG4996/Vps54L (Gillingham et al., 2014). However this protein was not detected in either dataset and so taken together these results suggest that Arl5 interacts with GARP but not with GARPII.

### Loss of Arl5 results in partial delocalization of a GARP complex subunit

**Fig. 4.**
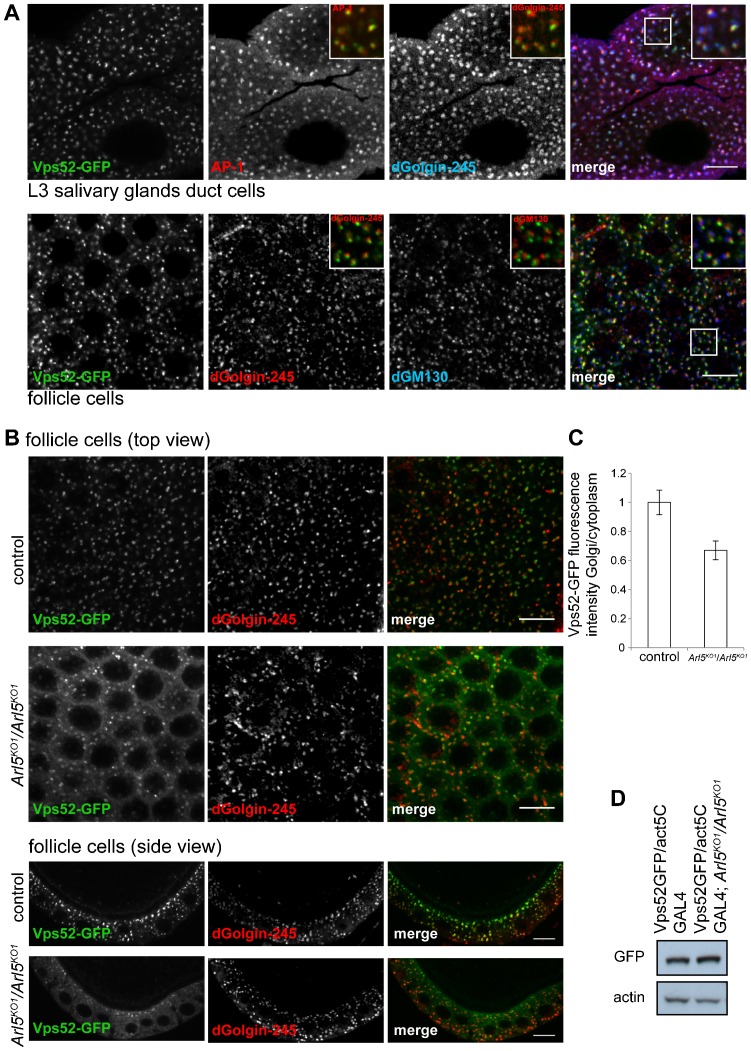
Arl5 is required for the normal localization of the GARP complex subunit Vps52 to the Golgi. (A) *Drosophila* Vps52 localizes to the TGN. Larval L3 salivary gland duct cells and epithelial follicle cells expressing Vps52-GFP under the control of Act5C promoter were stained for cis-Golgi and/or trans-Golgi markers. In duct cells, Vps52-GFP (green) was found adjacent to the trans-Golgi marker dGolgin-245 (blue) and partially overlapped with AP-1 (red) at the trans-Golgi network. In follicle cells, Vps52-GFP (green) was located next to dGM130 (blue) and juxtaposed to dGolgin-245 (red). The insets are magnifications of the indicated boxed areas. (B) Cross-section and side view of epithelial follicle cells from flies that were either homozygous for *Arl5^KO1^* mutation (*Arl5^KO1^*/*Arl5^KO1^*) or were *w1118* (control), expressing Vps52-GFP and stained for dGolgin-245. There is a decrease in the abundance and intensity of Vps52-GFP puncta in the *Arl5^KO1^* mutant, with a concomitant increase in the cytosolic pool. (C) Quantification of the effect of the Arl5 deletion as the ratio of the average fluorescence of Vps52-GFP at the Golgi in relation to the cytoplasmic pool of Vps52-GFP. A single image such as those in (B) was obtained from each of 11 flies for each stock, and the mean determined of the overall ratios of Golgi/cytoplasmic intensity in each image. Error bars show standard error of mean. The Golgi fraction of Vps52-GFP is 1.5 fold higher in wild-type follicle cells than in the *Arl5^KO1^*/*Arl5^KO1^* mutant, indicating that Arl5 aids the recruitment of the GARP complex to the Golgi. (D) The total amount of Vps52-GFP is comparable between control and the *Arl5^KO1^* mutant. Immunoblot of ovaries expressing Vps52-GFP in the presence (Vps52GFP/act5C GAL4) or absence of Arl5 (Vps52GFP/act5C GAL4; *Arl5^KO1^*/*Arl5^KO1^*) and probed for GFP and β-Actin, used as a loading control. Scale bars are 10 µm.

To verify whether Arl5 has a role in recruiting the GARP complex to the Golgi *in vivo*, we first generated a fly line expressing under UAS control a GFP-tagged form of the GARP subunit Vps52 (CG7371). In both salivary gland and follicle cells, Vps52-GFP was found in a punctate pattern typical of the Golgi, with the protein partially overlapping the trans-Golgi markers dGolgin-245 and AP-1, but adjacent to, and further from, the cis-Golgi marker dGM130. (Fig. 4A; supplementary material Fig. S1C). This implies that the exogenous Vps52-GFP is being incorporated into the GARP complex and that this tagged form of the complex is still recruited to the TGN. To test the requirement for Arl5 in the recruitment of Vps52-GFP we examined its distribution in control and in *Arl5^KO1^* mutant follicle cells. Removal of Arl5 reduced the intensity of Vps52-GFP on the Golgi and there was a substantial increase in the amount of GFP in the cytoplasm (Fig. 4B). This was confirmed by quantitation of the average fluorescence of the Golgi fraction of Vps52-GFP versus the cytoplasmic fraction (Fig. 4C), whilst protein blotting confirmed that the total amount of Vps52-GFP is similar between wild-type and *Arl5^KO1^* mutant ovaries (Fig. 4D). This indicates a deficit in the recruitment of the GARP complex to the trans-Golgi in the absence of Arl5.

### Arl5 loss results in enlargement of the endosomal compartment

**Fig. 5.**
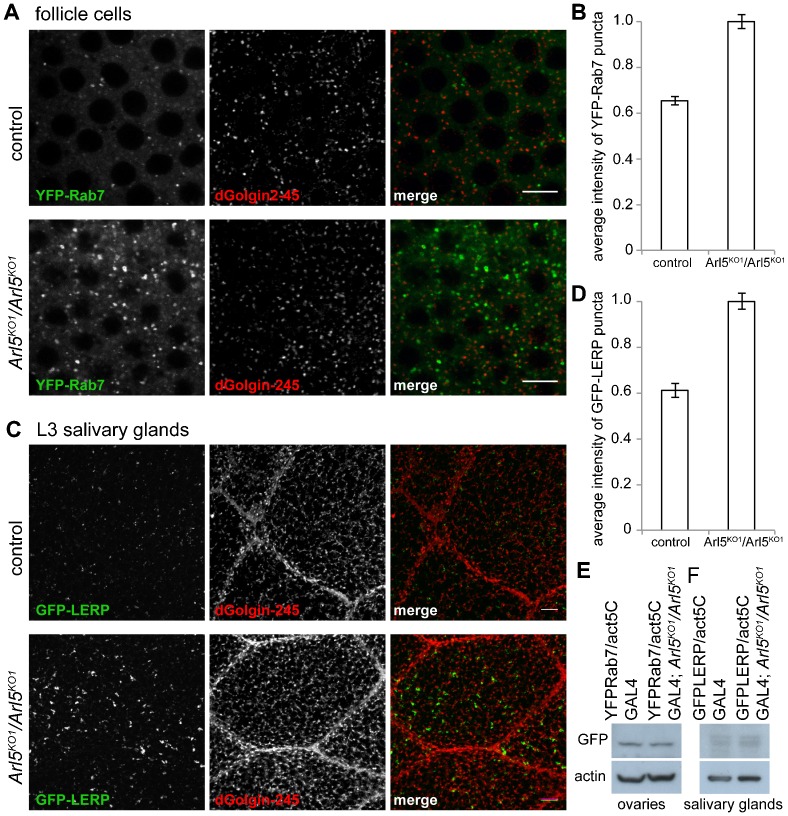
Loss of Arl5 leads to the accumulation of swollen endosomal compartments. (A) Cross-section view of epithelial follicle cells of control (*w1118*) and *Arl5^KO1^* mutant (*Arl5^KO1^*/*Arl5^KO1^*) shows that the loss of Arl5 results in the accumulation of enlarged late endosomal and lysosomal structures, marked by the exogenously expressed marker YFP-Rab7 (green). The trans-Golgi marker dGolgin-245 remains largely unaltered between mutant and control follicle cells. (B) Quantification of the extent of accumulation of YFP-Rab7-containing structures. A single image such as those in (A) was obtained from each of 13 flies for each stock, and a mean determined of average intensity of the cytoplasmic puncta in each image. Error bars show standard error of mean. The average fluorescence relative to that of cytoplasm being approximately 1.5× higher in the *Arl5^KO1^* homozygous mutant. (C) Confocal micrographs of L3 larval salivary glands from control (*w1118*) and *Arl5^KO1^* mutant (*Arl5^KO1^*/*Arl5^KO1^*), expressing a GFP-tagged form of the *Drosophila* receptor of precursor of lysosomal hydrolases (LERP), revealed that absence of Arl5 leads to the enlargement of structures positive for GFP-LERP (green), which only localized moderately with dGolgin-245 (red), both in control and in the *Arl5^KO1^* mutant. (D) Quantification of the extent of accumulation of GFP-LERP- containing structures. A single image such as those in (C) was obtained from each of 9 flies for each stock, and a mean determined of the average intensity of the cytoplasmic puncta in each image. Error bars show standard error of mean. The average fluorescence of GFP-LERP positive structures versus that of the cytoplasm is 1.6× higher in the *Arl5^KO1^* mutant than in control L3 salivary gland cells, suggesting its altered retrograde traffic from endosomes to the TGN. Scale bars are 10 µm. (E) Anti-GFP immunoblots of extracts from ovaries of flies that express YFP-Rab7 and either have, or lack, Arl5. (F) Anti-GFP immunoblots of extracts from salivary glands of flies that express GFP-LERP and either have, or lack, Arl5.

To further examine the consequences of the loss of Arl5 we analyzed the morphology of several compartments from the endocytic pathway by expressing in follicle cells the late endosomal marker Rab7 tagged with a fluorescent protein. We observed a clear enlargement of late endosomal and lysosomal structures in *Arl5^KO1^* mutant follicle cells as marked by YFP-Rab7 (Fig. 5A). Indeed, the average fluorescence of structures containing YFP-Rab7 is approximately 1.5 fold higher in the *Arl5^KO1^* mutant follicle cells (Fig. 5B). This phenotype was also observed in salivary glands after expression of YFP-Rab7 (supplementary material Fig. S2). To determine whether the enlargement of the YFP-Rab7 compartment was due to defects in retrograde transport from endosomes to the TGN we examined the distribution of GFP-LERP (lysosomal enzyme receptor protein), the *Drosophila* ortholog of the mammalian MPRs (Burgess et al., 2012; Hirst et al., 2009). In L3 larval salivary glands, structures positive for GFP-LERP were enlarged in the *Arl5^KO1^* mutant compared to the control (Fig. 5C), with an average fluorescence 1.6 fold higher relative to wild-type (Fig. 5D). Immunoblotting indicated that the altered distribution of Rab7 and LERP proteins was not a consequence of increased levels as these appeared unaffected by loss of Arl5 (Fig. 5E,F). Taken together, these results suggest that Arl5 loss leads to altered retrograde transport from endosomes and an enlargement of endosomal structures.

### Mammalian Arl5b is required for the recruitment of Vps54 to the TGN

We next examined whether a role for Arl5 in recruiting the GARP complex to the Golgi is conserved in humans. Mammals have two clear Arl5 paralogues, Arl5a and Arl5b that are 80% identical. A third gene, *ARL5C*, is a possible additional paralogue with 65–70% identity to the other two, but is perhaps more likely to be a pseudogene since there are no human or mouse ESTs for *ARL5C* in the NCBI database. In contrast, expression databases indicate that Arl5a and Arl5b are both expressed across the major tissues and anatomical regions with the expression levels of Arl5a being more variable in comparison to Arl5b. For each protein, siRNAs targeting four different sequences were tested against GFP fusions to Arl5a or Arl5b expressed in HeLa cells, and the one with the highest levels of knockdown was used thereafter (supplementary material Fig. S3A).

**Fig. 6.**
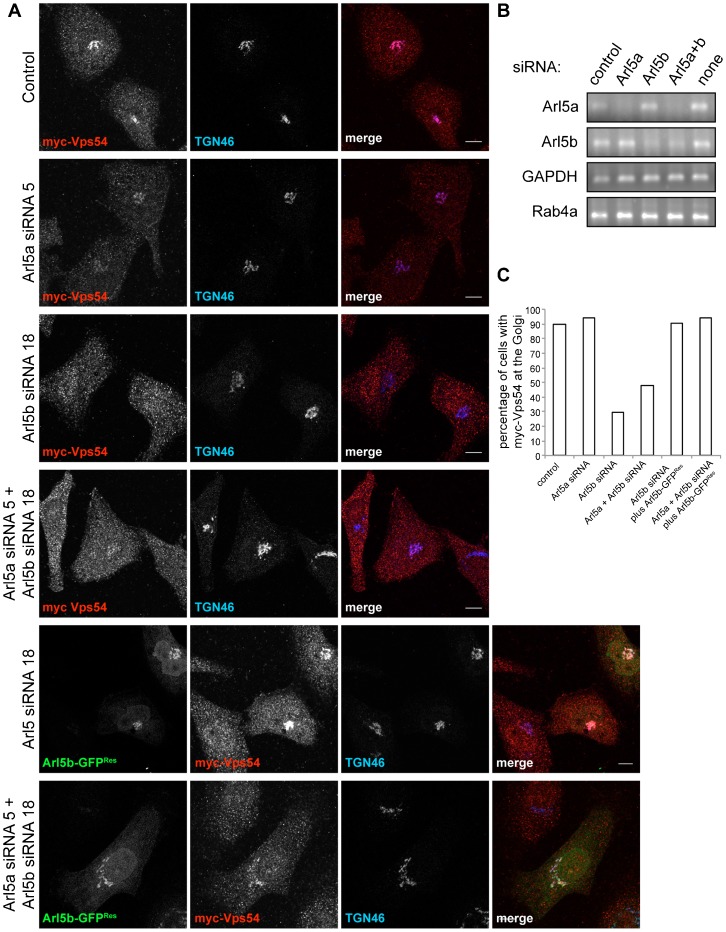
Arl5b is required for the recruitment of Vps54 to the TGN. (A) Confocal micrographs of HeLa cells expressing myc-Vps54 (red) and depleted of mock (control) or the indicated proteins with siRNAs and stained for TGN46 (blue). Knockdown of Arl5b or of Arl5a and Arl5b, simultaneously resulted in the striking redistribution of myc-Vps54 from the TGN to the cytoplasm. Expression of a siRNA-resistant form of Arl5b-GFP (Arl5b-GFP^Res^) was sufficient to rescue the mislocalization of myc-Vps54 upon depletion of either Arl5b or of Arl5a and Arl5b. Scale bars are 10 µm. (B) RT-PCR from HeLa cells silenced for the indicated proteins with siRNAs showed the efficient depletion of the corresponding mRNAs. Amplification of the housekeeping gene GAPDH and of Rab4a was used as a control for the total amount of mRNA across all samples. (C) Quantification of the effect of depletion of the proteins indicated in (A) on the localization of myc-Vps54. Transfected cells were examined for detectable myc-Vps54 on the Golgi (n = 29, 28, 47, 52, 32, 18 respectively). Strikingly, myc-Vps54 was redistributed from the TGN to the cytoplasm in 70% of cells silenced for Arl5b and in 52% of cells depleted for Arl5a and Arl5b. Expression of a siRNA-resistant form of Arl5b-GFP in cells depleted for Arl5b alone or for Arl5a and Arl5b increased the number of cells with myc-Vps54 at the TGN to levels comparable to mock silenced cells (control).

To monitor targeting of the GARP complex we used a myc-tagged version of Vps54, and consistent with previous studies (Pérez-Victoria et al., 2008), this was efficiently recruited to the Golgi in HeLa cells (Fig. 6A). RT-PCR from HeLa cells showed that both Arl5a and Arl5b are expressed, and confirmed efficient depletion of endogenous mRNA for either, or both simultaneously (Fig. 6B). Examining the distribution of myc-Vps54 showed that the knockdown of Arl5b, but not of Arl5a, resulted in a substantial loss of myc-Vps54 from the TGN and redistribution to the cytoplasm, with only 30% of cells analyzed maintaining myc-Vps54 at the TGN in contrast to 94% in the case of Arl5a depletion (Fig. 6A,C).

To verify that the cause of myc-Vps54 mislocalization was the loss of Arl5b, an siRNA-resistant form of Arl5b-GFP (Arl5b-GFP^Res^) was expressed in cells along with the siRNAs for Arl5b. Expression of Arl5b-GFP^Res^ in cells that lacked Arl5b resulted in the number of cells with myc-Vps54 at the TGN being increased to levels similar to the control, confirming the apparent requirement of Arl5b for efficient recruitment of the GARP complex to the TGN (Fig. 6A,C).

### Arl5a can substitute for Arl5b in promoting the recruitment of Vps54 to the TGN

The fact that knockdown of Arl5a did not affect the distribution of Vps54 may simply reflect a discrepancy between the expression levels of Arl5a and Arl5b in this particular cell type. A recent quantitative proteomic study of HeLa cells was able to detect Arl5b (estimated to be 6885 copies per cell), but could not detect Arl5a which suggests that even if it is expressed then the protein is at much lower levels than Arl5b (Kulak et al., 2014). Thus the relative effectiveness of knocking down Arl5b by itself may simply reflect that fact that it is the major form of Arl5 in this cell type. To determine if Arl5a can also contribute to the recruitment of GARP, we asked if Arl5a could rescue the Arl5b knockdown. When Arl5a-GFP was expressed in HeLa cells treated with Arl5b siRNA then it fully restored targeting of myc-Vps54 indicating that both proteins can direct GARP recruitment (supplementary material Fig. S3B,C).

## Discussion

The Arf small G proteins have emerged as a major family of regulators of membrane traffic and subcellular organization (Donaldson and Jackson, 2011). The first members to be characterized were Arf1 and Sar1 which recruit coat proteins and other effectors for membrane traffic to the ER and Golgi. However genome sequencing revealed the existence of a family of related or “Arf-like” proteins, several of which are conserved in non-metazoan eukaryotes such as plants and protozoa and seem likely to have fundamental roles in cellular organization (Burd et al., 2004; Gillingham and Munro, 2007; Kahn et al., 2006). The roles of these proteins have emerged over the last decade with Arl1 and ARFRP1 acting in membrane traffic at the trans-Golgi, Arl2 having a role in microtubule assembly, Arl3, Arl6 and Arl13 having a role in cilia and Arl8 controlling lysosomal motility and traffic. However Arl5 has remained perhaps the least well understood despite being conserved from mammals to plants. Localization studies revealed it to be on the Golgi, and ablation of function in mammalian tissue culture cells indicated a role in endosome to Golgi traffic (Gillingham and Munro, 2007; Houghton et al., 2012).

Our analysis of Arl5 in *Drosophila* was based on examining the phenotype of a null mutant, and affinity chromatography for effectors. Although flies lacking Arl5 are viable and fertile we could see alterations in the endosomal system consistent with sub-optimal traffic from endosomes to Golgi. The search for effectors revealed an interaction with the GARP complex that has been proposed to act in endosome-to-Golgi traffic, and the complex was partially delocalized from the Golgi in the absence of Arl5. This interaction appears likely to be conserved in mammals as we observed a similar effect on GARP localization when Arl5b was knocked down in human tissue cells, although we were unable to obtain biochemical evidence for specific binding of Arl5b to mammalian GARP, or any other protein. For reasons that are unclear we have found that small G proteins from *Drosophila* often work better for affinity chromatography than their mammalian orthologues (Gillingham et al., 2014).

The subunits of GARP have not been studied in depth in *Drosophila*, although mutations in Vps54 were identified in a screen for defects in spermatogenesis, and the gene was named *scattered* in reflection of the dispersed appearance of the spermatid nuclei (Castrillon et al., 1993). The molecular defect in the *scat^1^* allele has not been reported, and so it may not be a null allele. However given that it was noted to be semi-lethal, and that the Arl5 null is fertile, then it seems likely that loss of GARP has a more profound effect than loss of Arl5. This would be consistent with GARP being only partially displaced from the Golgi in the absence of Arl5, suggesting that there is some remaining GARP function mediated by other factors that recruit the complex to the Golgi. The viability of *Drosophila* lacking Arl5 is likely to reflect the fact that cells can apparently tolerate partial loss of function in retrograde traffic from endosomes to Golgi. Thus both yeast and murine ES cells are viable in the absence of GARP (Conibear and Stevens, 2000; Sugimoto et al., 2012). Loss of Vps54 results in embryonic lethality in mice, but in *C. elegans* GARP null mutants are viable (Luo et al., 2011; Schmitt-John et al., 2005). In many cases reduced or ablated GARP activity is associated with the accumulation of material in endosomes and the swelling of these compartments. Even if this does not cause cell death, it is likely to affect organism fitness. Future studies aimed at understanding the function of GARP and the related GARPII complex in the diverse cell types of complex metazoans should reveal more about how it contributes to membrane traffic, and thus what particular aspects of GARP function are controlled by Arl5.

## Supplementary Material

Supplementary Material
