# Supplementary material for: The small G protein Arl5 contributes to endosome-to-Golgi traffic by aiding the recruitment of the GARP complex to the Golgi
Source: Biol Open. 2015 Mar 20;4(4):474–81. doi: 10.1242/bio.201410975 (PMC4400590; doi:10.1242/bio.201410975)
Supplement: Supplementary Material [file supp_4_4_474__index.html]

The small G protein Arl5 contributes to endosome-to-Golgi traffic by aiding the recruitment of the GARP complex to the Golgi — The small G protein Arl5 contributes to endosome-to-Golgi traffic by aiding the recruitment of the GARP complex to the Golgi — Supplementary Material 

# The small G protein Arl5 contributes to endosome-to-Golgi traffic by aiding the recruitment of the GARP complex to the Golgi

## bio.201410975 Supplementary Material

**Files in this Data Supplement:**

- Supplementary Material - Cláudia Rosa-Ferreira et al. doi: 10.1242/bio.201410975
- Table S1 - **Mass spectrometric analysis of proteins bound to Arl5-coated beads Proteins identified as associating with Arl5-GDP or Arl5 GTP bound to beads.** The total spectral counts shown, and for each protein the gene number, protein name, FlyBase gene number (FBgn), and molecular weight (kDa) are stated.
- Table S2 - **Comparison of GTP-specific hits from bead and liposome-based affinity purification.** The top 20 proteins found bound to just the GTP-locked form of Arl5 in each data set as ranked by total spectral counts. For each protein the gene number, protein name, and molecular weight (kDa) are stated.
