# Supplementary material for: The small G protein Arl5 contributes to endosome-to-Golgi traffic by aiding the recruitment of the GARP complex to the Golgi
Source: Biol Open. 2015 Mar 20;4(4):474–81. doi: 10.1242/bio.201410975 (PMC4400590; doi:10.1242/bio.201410975)
Supplement: Supplementary Material [file supp_bio.201410975_bio.201410975-s1.pdf]

Biology Open (2015) 000, 1–8 doi:10.1242/bio.201410975

Supplementary Material

Cláudia Rosa-Ferreira et al. doi: 10.1242/bio.201410975

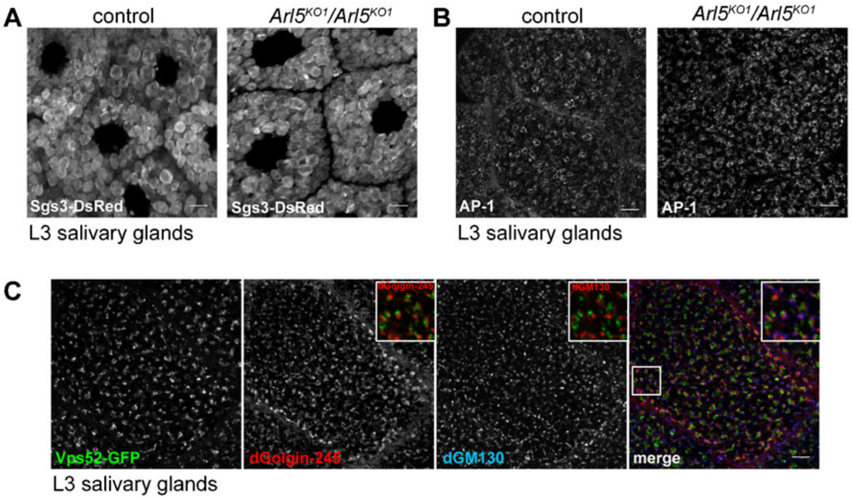

Fig. S1. Arl5 is not required for AP-1-dependent secretory granule formation. (A) Confocal micrographs of control (*w1118*) and of the *Arl5*^{KO1} mutant (*Arl5*^{KO1}/*Arl5*^{KO1}) mid/late L3 larval salivary glands expressing the glue protein Sgs3-DsRed. There were no obvious discrepancies in the number and size of secretory granules, marked by Sgs3, between control and the *Arl5*^{KO1} mutant. (B) Arl5 is not required for AP-1 recruitment, necessary for glue granule biogenesis. Endogenous AP-1 is found around glue granules in both control (*w1118*) and *Arl5*^{KO1} mutant (*Arl5*^{KO1}/*Arl5*^{KO1}) L3 larval salivary glands. (C) Confocal micrographs of L3 larval salivary glands show that the GARP complex, marked by the exogenous expression of Vps52-GFP, localizes to the TGN as it is found next to dGolgin-245 (red) and further apart from dGM130 (blue). Insets correspond to the boxed area, with Vp52-GFP in green and the indicated markers in red. Scale bars are 10 µm.

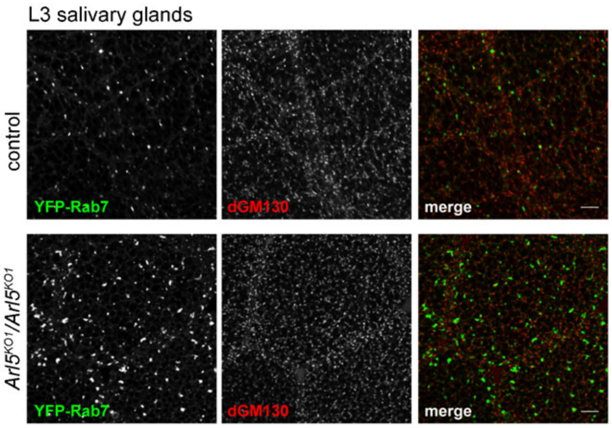

Fig. S2. Loss of Arl5 leads to the accumulation of swollen lysosomes in L3 larval salivary glands. Similarly to what has been observed in epithelial follicle cells, loss of Arl5 (*Arl5*^{KO1}/*Arl5*^{KO1}) in L3 larval salivary glands results in the accumulation of swollen structures positive for YFP-Rab7 (green), in comparison to control salivary glands (*w1118*). The cis-Golgi, represented by dGM130 (red) remains unaffected by the loss of Arl5. Scale bars are 10 µm.

Biology Open (2015) 000, 1–8 doi:10.1242/bio.201410975

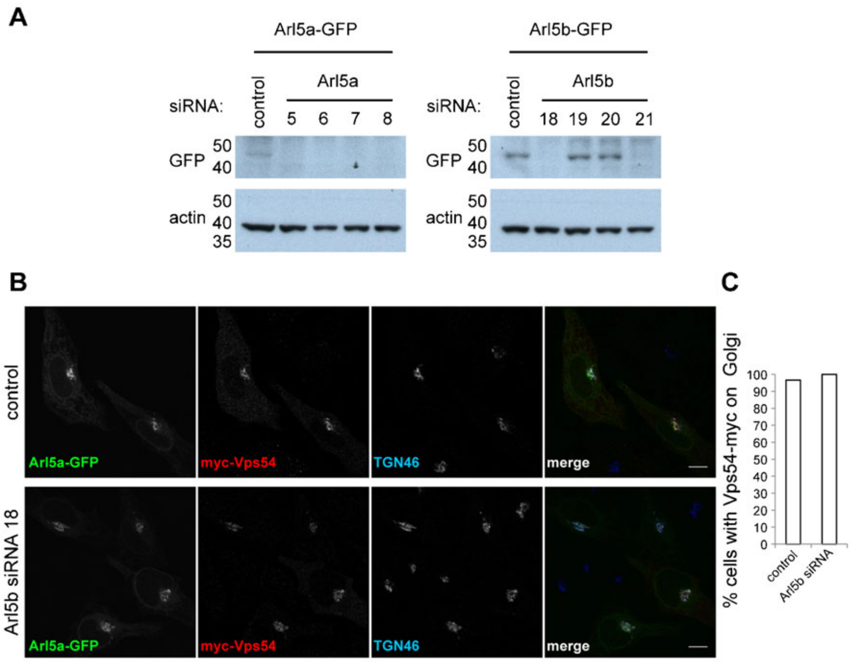

Fig. S3. Depletion of Arl5a or Arl5b by siRNA.
(A) Immunoblots of HeLa cells lysates expressing Arl5a-GFP or Arl5b-GFP and silenced for mock (control) or the indicated proteins (Arl5a or Arl5b) targeted by four different siRNAs each (5 to 8 for knockdown of Arl5a and 18 to 21 for Arl5b). The knockdown levels of the referred transgenes were evaluated by the decrease in the GFP bands, corresponding to either Arl5a-GFP or Arl5b-GFP, assuming equal amounts of total proteins for each sample by probing for β-Actin. (B) Confocal micrographs of HeLa cells expressing Arl5a-GFP (green) and myc-Vps54 (red) and depleted of mock (control) or Arl5b with siRNAs. After fixation the cells were stained for the myc-tag and TGN46 (blue). Expression of Arl5a-GFP was sufficient to rescue the mislocalization of myc-Vps54 upon depletion of Arl5b. Scale bars are 10 µm.
(C) Quantification of the effect on the localization of myc-Vps54 of the rescue by Arl5a-GFP of Arl5b siRNA.

Table S1: See supplementary webpage

Table S2: See supplementary webpage

Biology Open
